# Apparent absence of *Batrachochytrium salamandrivorans* in wild urodeles in the United Kingdom

**DOI:** 10.1038/s41598-019-39338-4

**Published:** 2019-03-12

**Authors:** Andrew A. Cunningham, Freya Smith, Trevelyan J. McKinley, Matthew W. Perkins, Liam D. Fitzpatrick, Owen N. Wright, Becki Lawson

**Affiliations:** 1 Institute of Zoology, Zoological Society of London, Regent’s Park, London, NW1 4RY UK; 2 Present Address: National Wildlife Management Centre, Animal and Plant Health Agency, Woodchester Park, Gloucestershire, GL10 3UJ UK; 3 University of Exeter, Penryn, Cornwall TR10 9FE UK; 4 Present Address: University of Exeter, Exeter, Devon EX4 4QD UK

## Abstract

Whether an infectious disease threat to wildlife arises from pathogen introduction or the increased incidence of an already-present agent informs mitigation policy and actions. The prior absence of a pathogen can be difficult to establish, particularly in free-living wildlife. Subsequent to the epidemic emergence of the fungus, *Batrachochytrium salamandrivorans* (Bsal), in mainland Europe in 2010 and prior to its detection in captive amphibians in the United Kingdom (UK), we tested archived skin swabs using a Bsal-specific qPCR. These samples had been collected in 2011 from 2409 wild newts from ponds across the UK. All swabs were negative for Bsal. Bayesian hierarchical modelling suggests that Bsal was absent from, or present at very low levels in, these ponds at the time of sampling. Additionally, surveillance of newt mortality incidents, 2013–2017, failed to detect Bsal. As this pathogen has been shown to be widespread in British captive amphibian collections, there is an urgent need to raise awareness of the importance of effective biosecurity measures, especially amongst people with captive amphibians, to help minimise the risk of Bsal spreading to the wild. Continued and heightened wild amphibian disease surveillance is a priority to provide an early warning system for potential incursion events.

## Introduction

The chytrid fungus, *Batrachochytrium salamandrivorans* (Bsal) is a recently-discovered, emerging pathogen causing epidemic mortality and population declines of urodele amphibians (newts and salamanders) in The Netherlands, Belgium and Germany^1,2^. The detection of Bsal in archived specimens exclusively from Asia and its widespread presence in China and Vietnam in the absence of disease, suggests that this pathogen is likely to have originated from Asia and was recently introduced into Europe^2–4^. In addition to the index site of emergence in Europe in a population of wild fire salamanders (*Salamandra salamandra*) on the Belgium/Netherlands border, the pathogen has recently been identified in wild amphibians in Germany^5^. Whereas no evidence of Bsal infection has been found on screening captive pet salamanders in the United States of America^6^, or on limited testing in Canada^7^, the pathogen has been detected in urodeles sampled from zoological and hobbyist collections in Europe, including in the United Kingdom (UK)^8–10^.

Although this newly-discovered pathogen can infect anurans, it is only known to cause disease in urodeles, albeit with huge variance in infection outcome amongst species, ranging from tolerance to death^2,11^. There are three native species of urodele in the UK: the great crested newt (*Triturus cristatus*), the smooth newt (*Lissotriton vulgaris*) and the palmate newt (*Lissotriton helveticus*). Whilst all three newt species are present across Great Britain (i.e. England, Scotland and Wales), only the smooth newt is present in Northern Ireland. The great crested newt is a Biodiversity Action Plan priority species, is listed on Appendix II of the Bern Convention and on Annexes II and IV of the EU Natural Habitats Directive, and is known to be susceptible to lethal infection with Bsal^2,11^. Infection with Bsal has been documented in smooth newts at multiple sites in mainland Europe^5^. In contrast, experimental challenge of the palmate newt with Bsal demonstrated resistance to infection in this species^2^. The alpine newt (*Ichthyosaura alpestris*), which is susceptible to fatal chytridiomycosis following experimental challenge with Bsal, and which has been found to be infected in the wild in mainland Europe^2,5^, is an invasive species in the UK with naturalised populations widely distributed across England and in parts of Scotland and Wales^12^. As all wild urodeles in the UK are newts, we use the latter term from here on to refer to these species.

In this study, we capitalise on existing sample archives and on disease surveillance to evaluate the likelihood of Bsal presence in wild newts in the UK, with the aim of informing mitigation strategies and contingency planning to prevent the incursion and impact of this pathogen on UK wildlife.

## Methods

### Skin swab sample collection

A survey was conducted in 2011 to investigate the spatial distribution of *B. dendrobatidis* (Bd) infection in wild amphibians in the United Kingdom^13^. Ponds were opportunistically selected to obtain a wide geographical coverage across the UK. As far as possible, sampling effort was stratified by region, with the number of sampled ponds commensurate with county size; easily accessible ponds were often preferentially selected. Non-invasive skin swabs were collected in the field by trained herpetologists and field ecologists and were tested in the laboratory for Bd using qPCR^13^. This study was approved by the Zoological Society of London’s Ethics Committee (WLE534) and all methods were performed in accordance with the relevant guidelines and regulations. Extracted DNA samples were archived at −80 °C and were consequently available for the current study. All available DNA extracts from both native and introduced newt species were tested using Bsal-specific qPCR as described by Blooi *et al*.^14^.

### Bayesian hierarchical model

The results of the Bsal qPCR analyses were pooled across all four newt species and were used to develop a Bayesian hierarchical model to estimate the infection prevalence in newts and ponds in the UK, as follows:

Let ${Y}_{i}$ be the number of positive swabs in pond $i$ ($i=1,\ldots ,P$), where $P=103$ is the number of ponds. Similarly, let ${X}_{i}$ be the number of newts sampled in pond $i$. To capture whether ponds are infected or not, we introduce a latent variable ${Z}_{i}$, such that

$${Z}_{i}=\{\begin{array}{cc}0 & {\rm{if}}\,{\rm{pond}}\,i\,\mathrm{is}\,\,\mathrm{uninfected},\\ 1 & {\rm{otherwise}}{\rm{.}}\end{array}$$

The number of positive swabs is then modelled as:

$${Y}_{i} \sim {\rm{Bin}}({X}_{i},{p}_{i}),$$

where

$${p}_{i}={Z}_{i}{p}_{i}^{D}{p}^{{\rm{sens}}},$$

**Figure 1:**
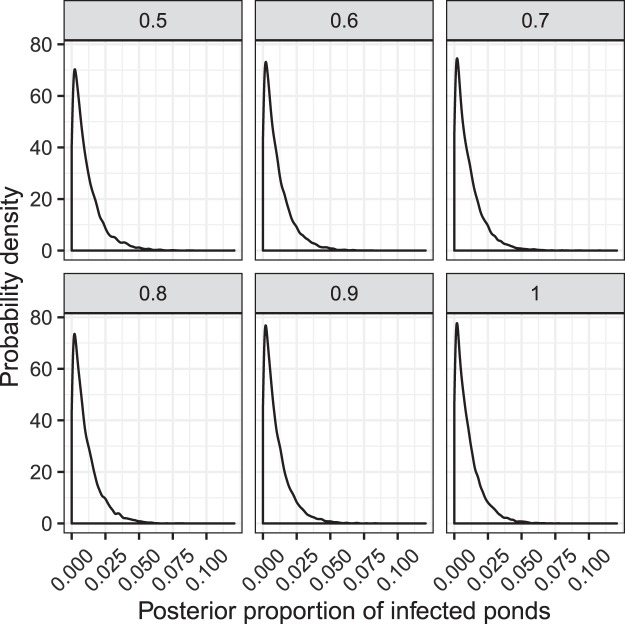
Posterior distributions for the mean prevalence of infected ponds. Facets denote different assumptions for the sensitivity of the diagnostic test.

**Figure 2:**
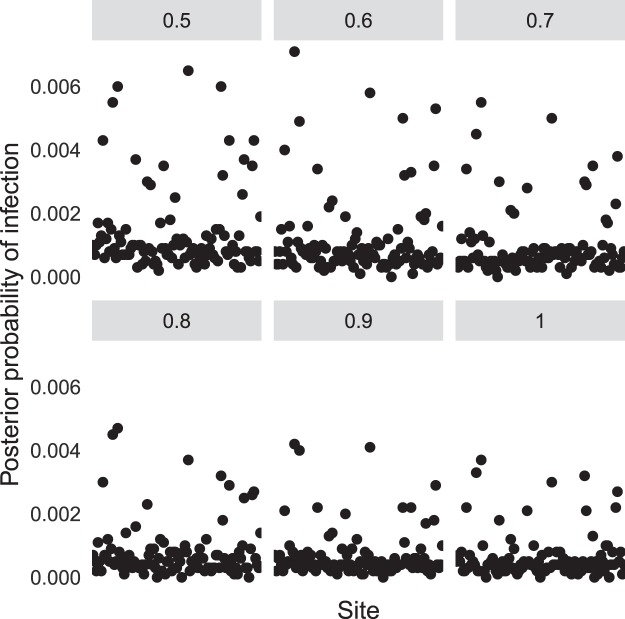
Posterior probability of infection for individual ponds. Facets denote different assumptions for the sensitivity of the diagnostic test.

${p}_{i}^{D}$ is the prevalence of the pathogen in pond $i$, given that pond $i$ is infected, and ${p}^{{\rm{sens}}}$ is the sensitivity of the diagnostic test (we assume 100% specificity here^15^). To complete the Bayesian specification, we use the following prior distributions

$$\begin{array}{c}{Z}_{i} \sim {\rm{Bern}}(p),\\ {p}_{i}^{D} \sim U(0,1),\\ p \sim U(0,1),\end{array}$$

where $p$ is the proportion of ponds that are infected. This approach allows us to estimate the marginal posterior distribution for the key parameter $p$, which integrates across the other sources of uncertainty (Fig. 1). We used vague prior distributions for ${p}_{i}^{D}\,$and $p$ to be conservative in our estimates. The posterior mean for each ${Z}_{i}$ provides an estimate of the posterior probability that pond $i$ was infected (see Fig. 2). Due to identifiability constraints, it was necessary to fix the sensitivity parameter ${p}^{{\rm{sens}}}$, and as such we decided to fit the model to a range of values, corresponding to $\,{p}^{{\rm{sens}}}\,$= 0.5, 0.6, 0.7, 0.8, 0.9 and 1. Lower values of ${p}^{{\rm{sens}}}\,\,$will result in higher false negative rates, which in turn allows for larger estimates of ${p}_{i}^{D}$ and $p$. From the literature we expect ${p}^{{\rm{sens}}}$ to be much larger than 0.5 (>0.9 even being conservative^15^). Of course, there may be some differences between this laboratory experiment and field sampling, and hence we explore a range of options, but for which we think that the lower bound of these choices will correspond to a highly conservative estimate for $p$.

Bayesian hierarchical modelling is a flexible framework, and our model is similar, in essence, to more complex occupancy models^16^, in the sense that it uses hierarchical structures to share information between different sampling sites. The complexity of our model is limited by the available data; the model could be extended, e.g. to analyse differences in infection prevalence between species, if more detailed data become available from future studies.

The data analysis and visualisations were conducted in R^17^, and the model was fitted using the freely available WinBUGS package^18^. All code necessary to repeat the analysis and to produce the figures and tables can be found in the Supplementary Methods. The raw data file can be found in the Supplementary Worksheet.

### Disease surveillance

Disease surveillance of wild amphibians was conducted across Great Britain, 2013–2017 inclusive. Reports of sick or dead amphibians, regardless of the perceived cause of illness or death, were solicited from members of the public. Carcasses were retrieved for post-mortem examination when the state of preservation permitted and these were examined using a systematic inspection protocol of the internal and external systems^19^. A post-mortem skin swab from each animal was examined using Bsal-qPCR.

## Results

### Retrospective analysis of skin swabs

Archived samples of extracted DNA from skin swabs collected in 2011 from 2409 newts at 103 sites (Table 1; Supplementary Worksheet) were available for the retrospective detection of Bsal. All three native newt species (*L. vulgaris*, *L. helveticus* & *T. cristatus*) and one invasive species (*I. alpestris*) were sampled; all tested qPCR negative for Bsal.

**Table 1:** Species tested for *Batrachochytrium salamandrivorans* using archived DNA extracted from skin swabs collected across the United Kingdom in 2011.

Species	Sex			Age		Total	No. of sites	No. of positive samples
	Male	Female	Unknown	Adult	Juvenile			
*Lissotriton vulgaris*	417	349	18	765	19	784	69	0
*Lissotriton helveticus*	655	484	22	1138	23	1161	63	0
*Triturus cristatus*	149	136	16	279	22	301	40	0
*Ichthyosaura alpestris*	81	74	8	144	19	163	12	0
All species	1302	1043	64	2326	83	2409	103	0

Using our Bayesian hierarchical model we explored the likely range of values for the posterior probability that any randomly selected pond was infected (or alternatively, an estimate of the posterior proportion of infected ponds). This is given by the parameter $p$ in the model specification. The Bayesian framework produces a distribution of possible outcomes for different test sensitivities (Fig. 1). From these distributions we derived point estimates and uncertainty bounds by calculating the posterior means and the area of highest posterior density (HPD) intervals (Table 2). These HPD intervals correspond to intervals between which we are 95% confident that the true value of the parameter lies. We also explored the posterior probabilities of infection for each individual pond, which are derived from the posterior means for the ${z}_{i}$ terms and are shown in Fig. 2.

**Table 2:** Posterior means and 95% highest posterior density (HPD) intervals for the proportion of infected ponds at different sensitivities of detection of *Batrachochytrium* *salamandrivorans*.

Sensitivity	Mean	Lower HPD	Upper HPD
0.5	0.011	3.4e-06	0.033
0.6	0.01	3.2e-07	0.031
0.7	0.011	2.5e-08	0.031
0.8	0.01	1.1e-06	0.031
0.9	0.01	3.2e-07	0.030
1	0.01	5.1e-06	0.030

Since we did not find any Bsal-positive swabs, the differences in the estimated posterior probabilities of infection for individual ponds correspond to differences in the number of samples analysed, with larger estimated values corresponding to ponds with low numbers of samples, which in turn corresponds to a higher probability of missing infection if it was indeed present in that pond. For ponds where smaller numbers of samples were taken, there is more uncertainty as to whether a pond was infected or not, which translates to higher estimates of the posterior probabilities of infection.

These results suggest that even with highly conservative estimates of the test sensitivity, the available data provide strong evidence that the proportion of Bsal-infected ponds is, at worst, very small (a 95% posterior probability that the proportion of infected ponds in the population is smaller than 3.3%). The use of vague prior distributions also means that the uncertainties in the posterior distributions will be larger than if we used more informative prior distributions, adding more weight to our conclusions that Bsal is unlikely to have been present in any of the ponds sampled, or if present at all, only present at very low levels. The posterior mean estimate for the proportion of infected ponds in the population is 1.1% in the worst-case scenario, and within the individual ponds sampled the largest posterior probability of infection was around 0.7%.

### Disease surveillance

We received 60 reports of wild newt mortality (+/− morbidity) from 40 sites in Great Britain, March 2013–December 2017 (mean of 1.6 dead newts per site, range 1–10). Of these, 43 animals (32 *L. vulgaris*, 7 *T. cristatus*, 1 *I. alpestris* and 3 unidentified species) from 18 sites were examined post mortem and their skin swabs were analysed. None were qPCR-positive for Bsal.

## Discussion

It is unusual to be able to examine such a large and relevant dataset prior to the detection of an emerging infectious disease in free-living wildlife. Our results demonstrate the value of disease and pathogen surveillance^20^ and of archiving samples^21^. While we found no evidence of Bsal infection in any of the 2409 samples collected from ponds sampled in 2011, it is not possible to conclude that the UK is free of Bsal in the wild because the available sample archive collected from 103 sites is just a subset of the newt population in the UK. Furthermore, pond selection was not random, and thus we must be careful when extrapolating our results to all ponds in the UK. Nonetheless, we did try to select ponds to give a wide spatial coverage. Indeed, one criterion for pond selection was accessibility^13^ and relatively frequent visitation compared to more remote ponds might be expected to increase the risk of exposure to Bsal, e.g. via contaminated fomites or the release of infected animals, and hence the likelihood of a study site testing positive for this pathogen. In combination with disease surveillance data from 2013–2017 (which also found no evidence of Bsal), our results indicate that Bsal is either not present in wild amphibians in the UK, or that its presence is localised and/or at low prevalence, consistent with a recent introduction. Given these results, for the purposes of disease mitigation and management strategy development, it is appropriate to adopt the precautionary principle and develop plans based on the assumption that wild amphibians in the UK are currently Bsal-free.

Modelling studies for Bsal in wild urodele populations in mainland Europe, where fire salamanders occur, suggest that disease outbreaks may occur at very low host population densities and can lead to the local extirpation of susceptible species within a few months^22^. Equivalent models do not yet exist for the UK. However, given that at least one UK native species (*T. cristatus*) is known to be susceptible to lethal infection with Bsal^2^, a strategy to prevent human-mediated introduction is a priority^23^. Crucially, this includes implementation of biosecurity measures^24^ within captive collections (to prevent introduction of Bsal from existing sources in the UK) and adequate quarantine and testing of imported amphibians (to avoid importing infection from new sources such as private collections or the amphibian trade). In addition, continued surveillance of wild populations is essential for early detection of incursion to enable mitigation protocols to be implemented in a timely manner to ensure maximum likelihood of success. Experience from mainland Europe, and efforts to develop treatment options for individuals and sites, should be used to help inform potential future control measures^10,25,26^.

In summary, whilst Bsal infection is known to be present in captive amphibians in the UK, we currently have no evidence of infection in free-living populations in this country. Since captive amphibians, including urodeles, continue to be imported into the UK^10^, the risk of incursion of this pathogen into UK wildlife is likely to continue. The European Commission recently implemented controls to prevent the spread of Bsal within the European Union (EU)^27^, but the small-scale trade of urodeles amongst hobbyists is unregulated and the introduction of costs and other hurdles associated with complying with the new EU regulations might direct more amphibian trade along unregulated pathways. Thus, raising the profile of Bsal within the hobbyist community and providing guidance on developing and maintaining Bsal-free collections is of key importance to minimising the risk of Bsal infection to both captive and free-living amphibians.

## Supplementary information

Supplementary Methods

Supplementary Worksheet

## Data Availability

All materials, data and associated protocols have been made available in the manuscript, Supplementary Methods and Supplementary Worksheet.
