# Supplementary material for: Apparent absence of Batrachochytrium salamandrivorans in wild urodeles in the United Kingdom
Source: Sci Rep. 2019 Mar 12;9:2831. doi: 10.1038/s41598-019-39338-4 (PMC6414544; doi:10.1038/s41598-019-39338-4)
Supplement: Supplementary file 1 — Supplementary Methods [file 41598_2019_39338_MOESM1_ESM.pdf]

Supplementary Methods: Apparent absence of *Batrachochytrium salamandrivorans* in wild urodeles in the United Kingdom.

Andrew A. Cunningham, Freya Smith, Trevelyan J. McKinley, Matthew Perkins, Liam Fitzpatrick, Owen N. Wright, and Becki Lawson

Introduction

This document describes a Bayesian model for estimating prevalences of *Batrachochytrium salamandrivorans* (Bsal) in newts in the United Kingdom, based on swab data obtained in 2011. This document (along with the corresponding data file), contain the code necessary to repeat the analysis in R[1] (using RStudio[2]) and the freely available WinBUGS package[3].

The analysis requires the `R2WinBUGS`[4], `coda`[5], `magrittr`[6] and `tidyverse`[7] suite of packages. This document was written using the `bookdown`[8] package, which is based on the core `rmarkdown`[9] and `knitr`[10] packages.

Firstly, we can load the necessary libraries. We assume throughout this document that WinBUGS has been installed in its default location and that all model and data files are in the same working directory in R. If not, then some amendments to the code will have to be made to ensure correct linkage to the necessary files.

```
## load libraries
library(R2WinBUGS)
library(coda)
library(tidyverse)
library(magrittr)
```

Data are provided as a Supplementary Data File (“counts.csv”) online. Now we read in and summarise the data.

```
## read in newt data
newtcounts <- read_csv("Supplementary Worksheet S1.csv") %>%
    mutate(Y = 0L) %>%
    select(SiteID, Y, X = `Total number of newts sampled`)
head(newtcounts)
```

```
# A tibble: 6 x 3
  SiteID      Y     X
  <chr>   <int> <int>
1 3           0    17
2 5           0    30
3 7           0    25
4 20          0     6
5 23          0    29
6 25          0     4
```

This has 103 ponds, with `SiteID` corresponding to an identifier for each pond, `Y` being the number of positive swabs per site, and `X` the number of swabs taken per site. We can summarise the data as follows:

```r
## produce a summary of the data
summary(newtcounts)
```

```
    SiteID                Y           X        
 Length:103         Min.   :0   Min.   : 2.00  
 Class :character   1st Qu.:0   1st Qu.:16.50  
 Mode  :character   Median :0   Median :29.00  
                    Mean   :0   Mean   :23.39  
                    3rd Qu.:0   3rd Qu.:30.00  
                    Max.   :0   Max.   :40.00  
```

Hence there are a range of sample sizes, due to the fact that the data were collected as part of an earlier study not focussed on Bsal. However, most ponds have at least 29 samples, with only a small number having very low numbers of samples. Of all the 2409 samples tested, none were positive for Bsal.

Bayesian model

Let Y_i be the number of positive swabs in pond i ($i = 1, \dots, P$), where $P = 103$ is the number of ponds. Similarly, let X_i be the number of newts sampled in pond i. To capture whether ponds are infected or not, we introduce a latent variable Z_i, such that

$$Z_i = \begin{cases} 0 & \text{if pond } i \text{ is uninfected,} \\ 1 & \text{otherwise.} \end{cases}$$

The number of positive swabs is then modelled as:

$$Y_i \sim \text{Bin}\left(X_i, p_i\right),$$

where

$$p_i = Z_i p_i^D p^{\text{sens}},$$

p_i^D is the prevalence of the disease in pond i, given that pond i is infected, and p^{sens} is the sensitivity of the diagnostic test (we assume 100% specificity here).

To complete the Bayesian specification, we use the following prior distributions

$$\begin{aligned} Z_i &\sim \text{Bern}\left(p\right), \\ p_i^D &\sim U(0, 1), \\ p &\sim U(0, 1), \end{aligned}$$

where p is the proportion of ponds that are infected. Due to identifiability constraints, we decide to fit the model to various fixed choices for the sensitivity of the test, p^{sens} (here we fit assuming values of 0.5, 0.6, 0.7, 0.8, 0.9 and 1).

WinBUGS code

This model can be described in WinBUGS using the following code (which needs to be saved to a new model file called 'model.txt' and stored in the working directory).

```
model
{
    for(i in 1:P)
    {
        ## probability that the pond is uninfected
        Z[i] ~ dbern(pinf)

        ## probability of getting a positive
        ## swab per pond = sens * prevalence
        pswab[i] <- Z[i] * pprevD[i] * psens

        ## probability of sampling positives
        Y[i] ~ dbin(pswab[i], X[i])
    }

    ## Priors
    pinf ~ dunif(0, 1)
    for(i in 1:P){
        pprevD[i] ~ dunif(0, 1)
    }
}
```

To run the model we choose to return posterior samples for p, p_i^D and Z_i, and run for different values of the sensitivity p^{sens}.

```
## function to randomly sample initial values
inits <- function(){
    list(
        pinf = runif(1, 0, 1),
        pprevD = runif(nrow(newtcounts), 0, 1)
    )
}

## set different sensitivities for running model over
sens <- seq(0.5, 1.0, by = 0.1)

## set output object
model_mcmc <- list()
```

```r
## loop over different sensitivities
for(i in 1:length(sens)) {
    ## set data
    bugs.data <- list(
        P = nrow(newtcounts),
        X = newtcounts$X,
        Y = newtcounts$Y,
        psens = sens[i]
    )

    ## sample initial values
    initials <- list()
    for(j in 1:2) {
        initials[[j]] <- inits()
    }

    ## call WinBUGS from R
    model_mcmc[[i]] <- bugs(
        data = bugs.data,
        inits = initials,
        parameters.to.save = c("pinf", "pprevD", "Z"),
        model.file = "model.txt",
        DIC = F,
        n.chains = 2,
        n.thin = 1,
        n.iter = 10000,
        n.burnin = 5000,
        working.directory = getwd(),
        clearWD = T
    )
}
```

Results

Proportion of infected ponds

A key output of the model is the posterior probability that any randomly selected pond is infected (or alternatively, the posterior proportion of infected ponds). We can produce a kernel density plot of the posterior distribution of p for different sensitivity values as:

```r
## extract post. mean prevalence of infected ponds
## and format into data frame to plot using ggplot
model_results <- model_mcmc %>%
    map("sims.matrix") %>%
    map(~{.x[, grep("pinf", colnames(.x))]}) %>%
    map(as.tibble) %>%
    bind_cols() %>%
    set_colnames(sens) %>%
    gather(sens, prev)
```

```r
## produce plot
p <- model_results %>%
    ggplot(aes(x = prev)) +
        geom_density() +
        facet_wrap(~ sens) +
        xlab("Posterior proportion of infected ponds") +
        ylab("Probability density") +
        theme_bw() +
        scale_x_continuous(breaks = c(0, 0.025, 0.05, 0.075, 0.1)) +
        theme(axis.text.x  = element_text(angle = 45, vjust = 0.5))
print(p)
```

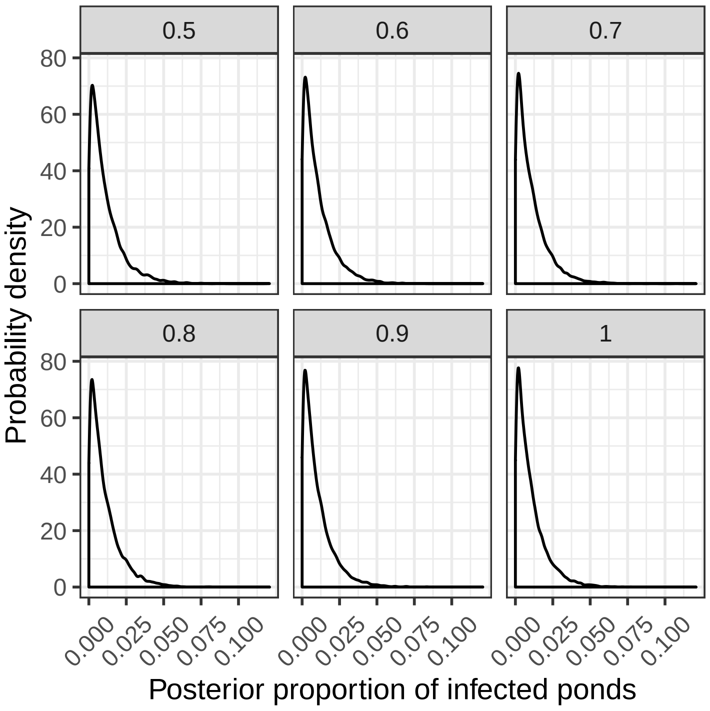

A table of posterior means and highest posterior density (HPD) intervals can also be calculated:

```r
model_results %>%
    group_by(sens) %>%
    summarise(
        mean = mean(prev),
        LHPD = HPDinterval(as.mcmc(prev))[1],
        UHPD = HPDinterval(as.mcmc(prev))[2]
    )
```

```
# A tibble: 6 x 4
  sens    mean          LHPD   UHPD
  <chr>  <dbl>         <dbl>  <dbl>
1 0.5   0.0109 0.00000345   0.0331
2 0.6   0.0104 0.000000320  0.0313
3 0.7   0.0105 0.0000000249 0.0309
4 0.8   0.0104 0.00000111   0.0310
5 0.9   0.0100 0.000000320  0.0300
6 1     0.0100 0.00000513   0.0300
```

Posterior probabilities of infection for individual ponds

We can calculate the posterior probabilities of infection for each pond by simply take the average of the corresponding Z_i samples for each pond as below. The larger estimated values are for those ponds with low numbers of samples, corresponding to a higher probability of missing infection if it was present in that pond.

```
## extract posterior samples for Z and amalgamate
## into correct form for plotting using ggplot
Z_results <- model_mcmc %>%
    map("sims.matrix") %>%
    map(~{.x[, grep("Z", colnames(.x))]}) %>%
    map(~{apply(.x, 2, function(x) sum(x) / length(x))}) %>%
    map(~{as.tibble(t(.x))}) %>%
    map(~{gather(., site, prob)}) %>%
    set_names(sens) %>%
    bind_rows(.id = "sens")

## produce plot (removing x-axis tick marks for clarity)
p <- Z_results %>%
    ggplot(aes(x = site, y = prob)) +
        geom_point() +
        facet_wrap(~ sens) +
        ylab("Posterior probability of infection") +
        xlab("Site") +
        theme(
            axis.text.x = element_blank(),
            axis.ticks = element_blank(),
            panel.background = element_blank()
        )
print(p)
```

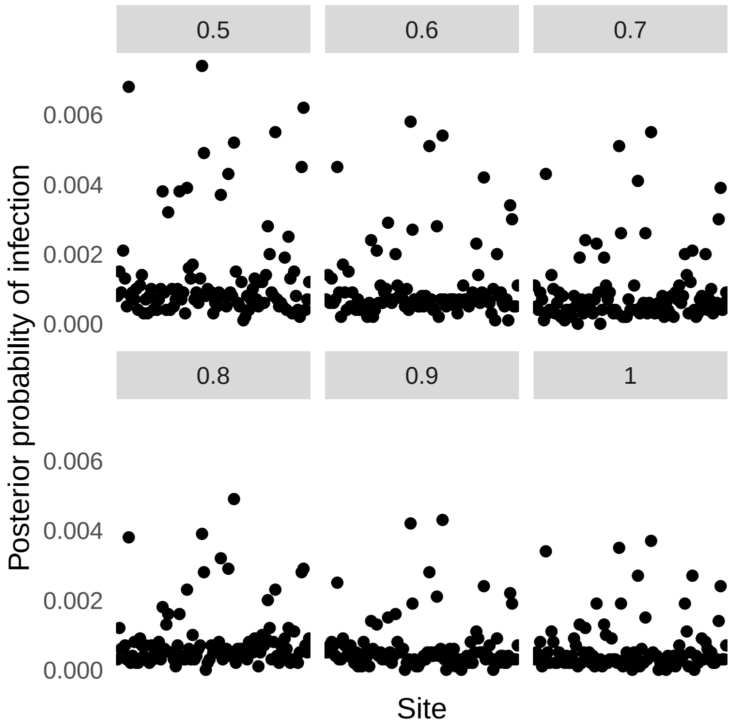

References

1. R Core Team. *R: A Language and Environment for Statistical Computing.* (R Foundation for Statistical Computing, 2016).

2. RStudio Team. *RStudio: Integrated Development Environment for R.* (RStudio, Inc., 2015).

3. Lunn, D. J., Thomas, A., Best, N. & Spiegelhalter, D. WinBUGS – a Bayesian Modelling Framework: Concepts, Structure, and Extensibility. *Statistics and Computing* **10,** 325–337 (2000).

4. Sturtz, S., Ligges, U. & Gelman, A. R2WinBUGS: A Package for Running Winbugs from R. *Journal of Statistical Software* **12,** 1–16 (2005).

5. Plummer, M., Best, N., Cowles, K. & Vines, K. CODA: Convergence Diagnosis and Output Analysis for Mcmc. *R News* **6,** 7–11 (2006).

6. Bache, S. M. & Wickham, H. *Magrittr: A Forward-Pipe Operator for R.* (2014).

7. Wickham, H. *Tidyverse: Easily Install and Load the 'Tidyverse'.* (2017).

8. Xie, Y. *Bookdown: Authoring Books and Technical Documents with R Markdown.* (Chapman; Hall/CRC, 2016).

9. Allaire, J. *et al.* *Rmarkdown: Dynamic Documents for R.* (2016).

10. Xie, Y. *Dynamic Documents with R and Knitr.* (Chapman; Hall/CRC, 2015).
